# Procrastination at the Core of Physical Activity (PA) and Perceived Quality of Life: A New Approach for Counteracting Lower Levels of PA Practice

**DOI:** 10.3390/ijerph17103413

**Published:** 2020-05-14

**Authors:** Nuria Codina, José V. Pestana, Rafael Valenzuela, Nuria Giménez

**Affiliations:** 1 Department of Social Psychology and Quantitative Psychology, University of Barcelona, 08035 Barcelona, Spain; ncodina@ub.edu (N.C.); rvalenzuela@ub.edu (R.V.); 2 Research Unit, Mútua Terrassa Research Foundation, University of Barcelona, 08221 Terrassa, Spain; ngimenez@mutuaterrassa.es; 3 Laboratory of Toxicology, Universitat Autònoma de Barcelona, 08193 Bellaterra, Spain

**Keywords:** adults, physical activity, procrastination, psychological benefits, quality of life

## Abstract

Faced with the demonstrated need to engage in physical activity (PA), lack of time is the argument commonly used to justify low or non-existent levels of PA. Underlying this argument, the accomplishment of procrastination behaviour seems to be related to the less time dedicated to practicing PA and the low perception of the quality of life. With this in mind, the purpose of this study is to show that dedicating different amounts of time to PA affects the perceived quality of life and the widespread problem of procrastination. We hypothesise that greater time investment in PA is related to greater perceived quality of life and less procrastination. In all, 621 practitioners of PA (347 men, 274 women) between 18 and 83 years old (*M* = 35.43, *SD* = 14.45) filled out validated versions of the World Health Organization quality of life assessment (WHOQOL-BREF) and the Pure Procrastination Scale. Results showed that people who do enough PA have a more positive perception of the quality of life in the domains of physical and psychological health; this perception, in turn, is related to lower levels of procrastination. Likewise, socio-demographic characteristics such as gender and the main activity presented significant associations with various quality of life domains and procrastination. In sum, the benefits of improvements in quality of life and reductions in procrastination identified in this study are sensitive to the time spent on PA, which suggests that a strategy to promote the practice of PA would improve time management and, thus, counteract procrastination.

## 1. Introduction

More than two-thirds of the inhabitants in the European Union practice little or no PA [1]. Specifically, in the case of the Spanish population, 34.4% do not engage in any PA, 38.9% practice insufficient PA, and the remaining 26.7% do enough PA, but mostly of moderate-intensity [2]. As a standard or parameter for adequate levels of PA, the World Health Organization (WHO) and the European Commission recommend that adults practice moderate physical activity (PA) for a minimum of 150 min per week, vigorous PA for 75 min/week, or a combination of the two [3,4].

This amount of PA has been found to have numerous benefits. For example, it has been found to improve the perception of the quality of life, even preventing and reducing problems such as type-2 diabetes, cardiovascular disease, hypertension, obesity, or cognitive impairment, as well as increasing life expectancy [5,6]. Likewise, from a more psychosocial perspective, it has been observed that the practice of PA is beneficial, given that it favours an optimistic outlook of life that makes it possible to deal with stress, anxiety, or the depressive state, improving feelings of control, competence, attention, self-esteem, self-image, or self-confidence [7,8]. The benefits of PA have been used as arguments for promoting it, but given that the incidence of its promotion is limited, it is important to enhance strategies for PA adherence and to analyse the causes behind the lack of its practice. In the investigation of the causes of insufficient PA practice, as mentioned above, various studies have highlighted that time scarcity has a negative effect on PA [9], justifying this insufficient PA practice with long workdays and low economic resources, in addition to family roles [9,10,11]. However, studies have shown that lack of time ceases to be a reason for not practicing PA when social support is available (parents, family, friends, or doctor) [12,13]. Moreover, research has shown that people who practice regular activities or have established routines that require a certain time commitment have a tendency to organise their time better and, consequently, complete their tasks within the periods and time required [14,15], which would include the practice of PA [16]. Consequently, it seems that the issue of time—and specifically lack of time—is not merely an objective question of time scarcity. 

From our point of view, underlying the argument of time scarcity, there is often the time management problem known as procrastination—a problem that affects different areas of daily life—which may ameliorate when PA is practiced regularly. Procrastination behaviour has been defined as an experience characterised by habitually—and often counterproductively—postponing the fulfilment of tasks [17]. The procrastination behaviour, in addition to being a problem that has a negative impact on completing tasks, also negatively affects the perception of the quality of life because it produces stress, anxiety, insomnia, and depression [18,19]. Although the phenomenon of procrastination has mainly been studied in academic settings [20,21,22,23], it has an influence on various aspects of everyday life [24,25], it tends to be somewhat more pronounced among men than among women but represents a problem throughout life, even though it gradually decreases with age [26,27]. Thus, it is estimated that half of all students and a fifth of adults see themselves as severe and chronic procrastinators [18]. However, the problem of procrastination related to poor time management decreases when there is a regular activity (work, studies, or other activities that are regularly present in the daily life of the person) requiring a certain amount of time and greater self-regulation of one’s time [14,15]. In turn, this self-regulation is associated with the perception of a better quality of life [28,29].

Related to this evidence about the importance of regularity in the practice of activities in the area of PA, some studies have been published on procrastination in relation to the initiation of sports practice [30,31]. In said studies, procrastination is considered as an independent variable that affects PA; however, it is also possible to consider procrastination as a dependent variable among those who practice PA with different intensities. Regarding the procrastination behaviour in those who practice PA, in agreement with studies on routines and regular activities, more or less time investment in PA would be expected to be associated with a higher or lower tendency to procrastinate and more or less self-management of time [32].

Based on the evidence described above, it can be assumed that insufficient dedication to the practice of PA, in addition to reducing aspects of quality of life, can also be associated with procrastination. In this study, we aim to show how different time investments in PA affect the practitioners’ perception of the quality of life and their time management—i.e., procrastination. Specifically, the following three study hypotheses are proposed:

**Hypothesis** **1** **(H1).** A greater time investment in PA (time spent per week and length of the sessions) is associated with higher perceived quality of life.

**Hypothesis** **2** **(H2).** A greater time investment in PA (time spent per week and length of the sessions) is associated with less procrastination.

**Hypothesis** **3** **(H3).** Better quality of life is associated with less procrastination.

Given the percentage of people who do not meet the minimum PA recommended, it is important to know to what degree the benefits vary depending on the time spent on physical activity. With this information, we could design better PA recommendation guidelines, offer reference data for the self-regulation of PA, and achieve a perception of a comfortable quality of life [33].

## 2. Materials and Methods 

### 2.1. Participants

Participants were 621 practitioners of PA (347 men and 274 women) ranging in age from 18 to 83 years old (*M* = 35.43 and *SD* = 14.45). Each practitioner was a user of one of eight municipal sports facilities in six districts of the city of Terrassa (Barcelona). These practitioners were interviewed when they were leaving these sports centres (i.e., after completing their PA). 

### 2.2. Instruments

Procrastination was measured with the Spanish version of the Pure Procrastination Scale [34], which consists of 12 items. There are five response options ranging from 1 to 5—from “does not describe me at all” to “very characteristic of me”—that stem from the Adult Inventory of Procrastination (“I find myself running out of time” [35]), the Decisional Procrastination Questionnaire (“I waste a lot of time on trivial matters before getting to the final decisions” [36]), and the General Procrastination Scale (“In preparation for some deadlines, I often waste time by doing other things” [37]). The instrument has shown an adequate Cronbach’s alpha in this study (0.92), higher than that of the validation mentioned above (α = 0.83 [33]), confirming the suitability of having followed recommendations [38] regarding the use of Spanish-version items of the Pure Procrastination Scale, previously validated [39].

To study the perceived quality of life, the instrument used was the abbreviated version—validated for our context—of the World Health Organization quality of life assessment (WHOQOL-BREF) [40,41]. This questionnaire, which is ideal for the analysis of health-related behaviours [42], includes 26 items with five response options. The items refer to the perception of the following domains: overall quality of life (“How would you rate your quality of life?”—one item), satisfaction with general health (“How satisfied are you with your health?”—one item), physical health (activities of daily living, energy, and work capacity—seven items, Cronbach’s α = 0.61), psychological health (body image, affectivity, self-esteem—six items, Cronbach’s α = 0.69), social relationships (social support, sexual activity—three items, Cronbach’s α = 0.67), and the environment (opportunities in the environment with regard to leisure, training, and health in general—eight items, Cronbach’s α = 0.65). 

In addition to the scales described above, socio-demographic information was obtained from the participants about their main activity (considering the categories of working, studying, unemployed, retired, and homemaker). In this way, the perception of the most characteristic time investment of daily life was determined, avoiding overlap between categories. Likewise, they were asked about their time investment in PA, expressed in time spent per week and the length of the sessions, each time they practiced physical activity. 

### 2.3. Procedure

The ethical requirements of the Ethics Committee of the University of Barcelona (University of Barcelona’s Bioethics Commission, CBUB—Institutional Review Board IRB00003099) were followed in the current study, which meant that additional approval for the research was not required because the data obtained did not involve animal or clinical experimentation. Additionally, this study complies with the recommendations of the General Council of Spanish Psychological Associations (Consejo General de Colegios de Psicólogos), the Spanish Organic Law on Data Protection [43], and the Declaration of Helsinki [44].

To carry out the present study, the sports centres designated for the practice of PA were selected in the city of Terrassa (Barcelona), and training was provided to the personnel responsible for collecting the information and obtaining the study data.

The sport centres were selected based on the municipal census of the Town Hall of the city of Terrassa. Specifically, publicly-owned facilities were chosen in each of the six districts of the city. Then, each facility was contacted to confirm its schedule and general user profile—ruling out, for example, times of the day when some sports centres receive school-aged children.

The people responsible for collecting the information were master’s degree students from one of the institutions involved in the study. They participated voluntarily; that is, no credits or other benefits from the interviews were offered as an incentive. These interviewers were prepared in one training session with the researchers, who also supervised the entire data collection process. The information obtained did not reveal any disparities that might be linked to the interviewers’ personal profiles or work styles.

The interviewers rotated their presence in the sports facilities, the three selected times of the day (mid-morning, late morning, and afternoon), and the five days allocated for data collection (Monday through Friday). The interviews, which lasted from 12 to 15 min, took place after the interviewee had practiced PA in the sports centres. Only those who agreed to participate in the study were interviewed, and rejections were not counted. The data collection process was completed in four weeks. No significant differences attributable to the selected sports facilities or the time of day (or day of the week) were observed in the information gathered, which means that no biases were observed that could be attributed to the data collection process. However, out of the 674 respondents who engaged with the interview, 53 responses had to be discarded due to defects in the registered information, mainly stemming from people who began to respond but afterwards claimed to be in a hurry and unable to finish answering.

The analysis of the data obtained included three procedures. First, descriptive analyses were conducted on the sociodemographic information considered, the time investment in PA, the perceived quality of life, and procrastination. Second, Pearson *r* correlations were conducted on the domains related to the quality of life and pure procrastination, and ANOVAs (or *t*-tests, depending on the case) were performed on the socio-demographic variables and the time spent on PA practice, with regard to the perceived quality of life factors and procrastination. Third, a regression analysis (stepwise) was carried out, with procrastination as the dependent variable and the time investment in PA and the perceived quality of life factors as independent variables. All the analyses described were conducted using SPSS (IBM SPSS Statistics, version 25, Armonk, NY, USA).

## 3. Results

The most common main activities of the users of the studied sport centres were working (60.7%) and studying (32.0%), with much lower percentages of participants who were retired (5.5%), unemployed (1.1%), and homemakers (0.6%). With regard to the time spent on PA, 69.3% practiced 150 min or more of moderate or intense PA per week (the remaining 30.7% practiced up to 150 min). With regard to the length of the sessions each time they performed physical activity, in 27.7% of the cases, sessions lasted less than 60 min, in 52.2%, sessions lasted between 60 and 119 min (that is, between one hour and almost two hours), and in the remaining 19.8%, sessions lasted 120 min or more.

In relation to the perceived quality of life (Table 1), results show that the psychological well-being domain is the one rated most positively (*M* = 4.00, *SD* = 0.51), and the domain of environmental well-being is the least positively rated domain (*M* = 3.74, *SD* = 0.47). In the case of procrastination (Table 1), the findings reveal that it lies below the midpoint (*M* = 2.34, *SD* = 0.72). Regarding the values of skewness and kurtosis (Table 1), all factors were non-normally distributed. The correlation of these scores (Table 1) shows that procrastination scores are inversely correlated with the quality of life domains related to general (*r* = −0.137, *p* < 0.001, physical (*r* = −0.234, *p* < 0.001), and psychological well-being (*r* = −0.185, *p* < 0.001).

The ANOVAs and *t*-tests based on socio-demographic variables, the time investment in PA (weekly time invested, length of the sessions), perceived quality of life factors, and procrastination (Table 2) point to significant differences based on gender, main activity, and time investment in PA. 

In the case of gender, these differences are observed in the quality of life domains of perception of general health (*t* = −2.10, *p* = 0.035, *d* = 0.13), and marginally in psychological well-being (*t* = −1.90, *p* = 0.057, *d* = 0.15). In both cases, men report higher scores than women. With regards to age, participants aged 18–29 years old show the highest values in the WHOQOL-BREF domains related to overall quality of life (*F* = 3.26, *p* = 0.039, *f* = 0.09) and general health (*F* = 4.64, *p* = 0.010, *f* = 0.14); these younger participants also show the highest scores in Pure Procrastination (*F* = 22.08, *p* = 0.000, *f* = 0.25). In addition, main activity is decisive in various quality of life domains (in parentheses, the categories with higher/ lower scores): General quality of life (students/unemployed; *F* = 3.97, *p* = 0.003, *f* = 0.16), overall quality of life (workers/unemployed; *F* = 9.70, *p* = 0.000, *f* = 0.36), physical health (workers/homemakers; *F* = 3.49, *p* = 0.008, *f* = 0.14), and social relationships (homemakers/unemployed; *F* = 3.89, *p* = 0.004, *f* = 0.15).

Regarding time dedicated to PA and quality of life indicators, it is worthwhile to distinguish between the weekly time investment in this activity and the length of the practice sessions. Results show that those who dedicate less than 150 min per week to PA perceive their quality of life related to the environment more positively (*t* = 3.21, *p* = 0.001, *r* = 0.13). With regard to the length of the sessions, those who engage in shorter sessions also have a more positive perception of the environment (*F* = 8.74, *p* = 0.000, *f* = 0.33), whereas those who engage in longer sessions score higher on the psychological health domain (*F* = 4.52, *p* = 0.011, *f* = 0.18).

Linear regression analyses were carried out to predict procrastination based on the dimensions of quality of life and weekly time investment in physical activity (Table 3), and multicollinearity was not deemed a problem (Durbin–Watson = 1.755) for any of the three significant predictors of procrastination (Physical health: Tolerance = 0.810; VIF = 1.235; Psychological health = Tolerance = 0.993; VIF = 1.007; Weekly time investment in PA = Tolerance = 0.809; VIF = 1.236). Of the models obtained, the third model (*R*^2^ = 0.09) shows that procrastination is influenced by perceptions of quality of life related to physical health (*β* = −0.20, *t*(609) = −4.83, *p* < 0.000) and psychological health (*β* = −0.08, *t*(609) = −1.97, *p* < 0.048), as well as the time dedicated to PA per week (*β* = −0.18, *t*(609) = −4.83, *p* < 0.000). 

With regard to the proposed hypotheses, the regression analysis also explains that the explanatory power of the weekly time investment in the case of procrastination. Likewise, it reveals that the physical and psychological health domains of perceived quality of life predict procrastination.

## 4. Discussion

Our study with PA practitioners shows that the percentage who dedicate more than 150 min per week to PA is slightly higher than what is found in the general population [1,2]. In this regard, it should be pointed out that the study was carried out with users of sports facilities, that is, people who decided to become associated with physical and human environments where PA is done, which may invite—or even pressure—them to dedicate more time to PA and enjoy more of its benefits. In this context where almost 70% of people dedicate 150 min or more to PA, the study reveals the robustness in the perceived quality of life domains, supporting recent studies by the WHOQOL-BREF in our context [40], as well as pointing out the need to deepen the structure and functioning of this instrument in future studies (due to the WHOQOL-BREF Cronbach’s alphas in this research). 

With regard to our first hypothesis—which assumed a direct relationship between the time investment in PA and the perceived quality of life—this assertion was confirmed in the case of the general health domain of perceived quality of life. Specifically, those who spend 150 min or more per week on PA have better perceived general health. By contrast, if more than 150 min/week are dedicated to PA or the sessions last more than 60 min, there is a perception of less quality of life in terms of opportunities for leisure, training, or safety in the environment. Therefore, dedicating more than 150 min to PA is associated with a more satisfactory perception of one’s general health and a less satisfactory perception of the opportunities in the environment. Due to what these findings could reveal—more PA time in sport centres as a response to the lack of opportunities or insecurity—they should be studied in greater detail, so that, in addition to showing significant associations like the ones observed here, they would show greater effect sizes. 

The second study hypothesis—that a greater time investment in PA is related to less procrastination—was fulfilled for both indicators of time spent on PA. Thus, those who practice PA more than 150 min per week or engage in sessions lasting at least one hour present lower levels of procrastination. These results coincide with findings showing that performing regular routines and activities that require a certain regular amount of time is a way to counteract procrastination [14,15,25]. Therefore, if spending more time on PA makes one procrastinate less in life in general, then greater dedication to PA is associated with more and better time management. With these results and their corresponding assessment, we show a benefit associated with PA that had not been demonstrated until now. Specifically, these benefits of PA present two axes: increasing the self-management of time and reducing procrastination and its adverse effects on quality of life. In particular, the relationship between PA and procrastination is an important finding, given the widespread problem of procrastination in advanced societies and the difficulties in correcting this problem, beyond individual therapeutic contexts [45,46].

The third hypothesis, proposing an inverse relationship between perceived quality of life and procrastination, was observed in almost all the domains of perceived quality of life (except overall quality of life, evaluated by the WHOQOL-BREF with only one item), but with small effect sizes. However, this study provides more detailed evidence about this relationship—even in people with levels of quality of life more than one point above the mean. The observed relationships also corroborate the incipient evidence in this line of research [47]. 

Even though all relationships among variables were not equally significant and had different effect sizes, our results show the importance of the weekly time investment in PA (but not the length of the sessions) and the perceived quality of life domains related to physical health (daily life activities, energy, and work capacity) and psychological health (body image, affectivity, self-esteem) in procrastination. Thus, more dedication to PA and a higher perception of the cited domains of quality of life tend to lead to lower procrastination levels. This result complements previous findings and further reinforces the idea of good time management as a variable related to a more positive perception of quality of life [28,29], as well as the importance of the time invested in PA. Consequently, if those who practice PA in the recommended amounts of time have better time management, in future studies, it is worth investigating to what extent those who do not practice PA do it due to poor time management. One of the significant differences observed in the present study that should be examined more in depth in future studies is related to gender and quality of life. The presence of these differences in a sample with a healthy lifestyle corroborates what has been observed in European samples over 50 years old [48], as well as the different strategies used by men and women to achieve better quality of life [49]. 

Finally, the analysis carried out in this sample of people who practice PA shows, in addition to what was proposed in the hypotheses, that sociodemographic characteristics such as gender, age and the main activity are associated with perceived quality of life and procrastination. In this regard, it is worth noting the gender differences, to the detriment of women, because men have higher scores on the general and psychological health domains, and no significant gender differences in procrastination as might have been expected. In the case of age, observing that procrastination is more present in the youngest, or, in other words, decreases in older age groups of the sample. With regard to the main activity, having a regular occupation has positive aspects because those who work and study rate their overall quality of life, general health, and physical health more positively, whereas homemakers stand out in their assessment of social relationships. Finally, not having a schedule seems to favour procrastination, as in the case of unemployed people and students [50].

The sample used in the study was obtained in sports facilities and, therefore, does not allow us to draw comparisons with people who are not physically active (the majority of the general population). This selection of participants could limit the generalisation of the results obtained; however, it guarantees a more homogeneous sample of PA practitioners because all of them have actively decided to practice PA, and they do not have a negative attitude toward it. Therefore, by working with a sample conducive to PA, we have raised an important issue related to the fact that even dedicating less time than recommended to PA, this dedication has beneficial effects, that is, the degree to which practicing PA less than the recommended time affects one of its most widely documented benefits (if the dedication is the recommended amount)—the quality of life. Another important issue related to the homogeneity of the sample is that, in people who do the recommended amount of activity, the procrastination behaviour lies below the mean.

With the results obtained by combining PA intensity, quality of life, and procrastination, this study reveals a new benefit associated with PA that runs in parallel with a better quality of life: less procrastination. Therefore, although these results should be contrasted in future studies (for example, with MANOVAs or other statistical procedures), our findings respond to the need to improve intervention strategies for the promotion of PA. As a matter of fact, this intervention is of special importance given the increase in procrastinators and procrastination practices in the course of academic life, especially at an early age. 

The correlational nature of the study is another limitation in terms of the precision in finding causal relationships among the variables. Therefore, future studies should test the relationships described in the results section. In these studies, it will also be necessary to introduce other variables that increase the percentage of explained variance and effect sizes of the reasons for procrastination. 

## 5. Conclusions

This study provides new knowledge about the benefits derived from PA, depending on the weekly time invested. Specifically, with regard to quality of life, when PA is practiced 150 min or more per week, the perception of general health is more positive; by contrast, when PA is practiced less than 150 min per week, the opportunities and alternatives offered by the environment are rated more positively. In addition, a new benefit associated with PA has been identified: the reduction in the widespread problem of procrastination, which might not be detected in the presence of an apparently unavoidable objective factor such as time scarcity, the usual argument for not doing the recommended levels of PA. Specifically, the results show that this problem declines when 150 min or more are dedicated to PA. However, when the dedication is less than 150 min per week, procrastination is higher, along with a low perceived quality of life in the domains of physical and psychological health. 

Because this study reveals new knowledge about the variability in some benefits of PA depending on the time dedicated to it, these benefits should be more closely examined in order to implement more effective public health interventions related to promoting the practice of PA, focusing on the role of procrastination in this practice. 

## Figures and Tables

**Table 1:** Means, standard deviations, and intercorrelations for domains related to quality of life and pure procrastination.

Domains Related to Quality of Life and Pure Procrastination	*M*	*SD*	Skewness	Kurtosis	1	2	3	4	5	6	7
1. Overall quality of life	30.86	00.72	−0.452	0.957	-						
2. General health	30.94	00.75	−0.501	0.384	0.436 **	-					
3. Physical health	30.95	00.42	−0.654	0.642	0.336 **	0.454 **	-				
4. Psychological	40.00	00.51	−0.508	0.587	0.398 **	0.408 **	0.434 **	-			
5. Social relationships	30.82	00.63	−0.393	0.360	0.270 **	0.278 **	0.329 **	0.482 **	-		
6. Environment	30.74	00.47	−0.392	0.628	0.409 **	0.299 **	0.442 **	0.372 **	0.343 **	-	
7. Pure procrastination	20.34	00.72	0.157	−0.142	−0.056	−0.137 **	−0.234 **	−0.185 **	−0.080 *	−0.093 *	-

* *p* < 0.01; ** *p* < 0.001.

**Table 2:** Descriptive statistics for domains related to quality of life and pure procrastination, with regard to socio-demographic characteristics and participation in physical activity.

Socio-Demographic Characteristics	Overall Quality of Life			General Health			Physical Health			Psychological			Social Relationships			Environment			Pure Procrastination		
	*M* ± *SD*	*F*	*p*	*M* ± *SD*	*F*	*p*	*M* ± *SD*	*F*	*p*	*M* ± *SD*	*F*	*p*	*M* ± *SD*	*F*	*p*	*M* ± *SD*	*F*	*p*	*M* ± *SD*	*F*	*p*
Gender		−1.66	0.097		−2.10	0.035		−1.83	0.072		−1.90	0.057		0.44	0.655		−0.27	0.978		−1.03	0.300
Men	3.91 ± 0.74			3.99 ± 0.76			3.98 ± 0.39			4.04 ± 0.50			3.81 ± 0.64			3.74 ± 0.46			2.37 ± 0.72		
Women	3.81 ± 0.69			3.86 ± 0.74			3.92 ± 0.45			3.96 ± 0.52			3.83 ± 0.62			3.74 ± 0.48			2.31 ± 0.71		
Age		3.26	0.039		4.64	0.010		1.51	0.220		1.09	0.335		2.63	0.073		1.72	0.179		22.08	0.000
18–29	3.95 ± 0.69			4.01 ± 0.77			3.96 ± 0.41			4.03 ± 0.54			3.89 ± 0.63			3.73 ± 0.44			2.55 ± 0.68		
30–49	3.81 ± 0.72			3.94 ± 0.72			3.98 ± 0.41			3.99 ± 0.48			3.78 ± 0.63			3.73 ± 0.49			2.22 ± 0.74		
50 and more	3.78 ± 0.79			3.75 ± 0.78			3.89 ± 0.43			3.95 ± 0.49			3.75 ± 0.62			3.82 ± 0.51			2.10 ± 0.62		
Main activity		3.97	0.003		9.70	0.000		3.49	0.008		1.36	0.244		3.89	0.004		2.14	0.074		10.65	0.000
Working	3.87 ± 0.68			4.00 ± 0.68			4.00 ± 0.40			4.02 ± 0.48			3.82 ± 0.63			3.79 ± 0.47			2.24 ± 0.69		
Studying	3.92 ± 0.72			3.93 ± 0.79			3.90 ± 0.42			3.99 ± 0.55			3.86 ± 0.61			3.67 ± 0.45			2.57 ± 0.73		
Unemployed	3.00 ± 1.15			2.42 ± 1.27			3.85 ± 0.47			3.64 ± 0.77			2.95 ± 0.84			3.59 ± 0.44			2.92 ± 0.81		
Retired	3.61 ± 0.92			3.61 ± 0.81			3.80 ± 0.45			3.91 ± 0.53			3.74 ± 0.59			3.73 ± 0.53			1.99 ± 0.60		
Homemaker	3.75 ± 0.95			3.75 ± 0.50			3.75 ± 0.65			3.87 ± 0.66			4.08 ± 0.31			3.61 ± 0.75			2.25 ± 0.22		
**Participation in Physical Activity**																					
Weekly frequency *		−0.01	0.987		−2.03	0.042		1.08	0.279		−1.21	0.226		1.193	0.234		3.21	0.001		4.81	0.000
Up to 150 min	3.86 ± 0.64			3.85 ± 0.67			3.98 ± 0.40			3.97 ± 0.48			3.86 ± 0.57			3.83 ± 0.44			2.54 ± 0.66		
150 min or more	3.86 ± 0.76			3.97 ± 0.79			3.94 ± 0.42			4.02 ± 0.52			3.80 ± 0.65			3.71 ± 0.48			2.25 ± 0.73		
Session length		0.29	0.748		0.97	0.377		1.12	0.327		4.52	0.011		1.23	0.291		8.74	0.000		6.57	0.002
Less than 60 min	3.88 ± 0.62			3.90 ± 0.66			3.97 ± 0.41			3.99 ± 0.45			3.85 ± 0.55			3.87 ± 0.40			2.51 ± 0.66		
60 min–119 min	3.84 ± 0.70			3.92 ± 0.75			3.97 ± 0.40			3.96 ± 0.53			3.78 ± 0.65			3.69 ± 0.48			2.29 ± 0.73		
120 min or more	3.90 ± 0.89			4.02 ± 0.89			3.90 ± 0.48			4.13 ± 0.51			3.87 ± 0.66			3.71 ± 0.51			2.24 ± 0.75		

Mean ± standard deviation. * *t* statistic reported for these variables.

**Table 3:** Regression analysis summary for domains related to quality of life and participation in physical activity predicting procrastination.

Model	*B*	*SE B*	95% CI	*β*	*t*	*p*	*R* ^2^
Model 1:							0.05
Physical health	−0.40	0.06	(−0.53, −0.27)	−0.23	−50.99	0.000	
Model 2:							0.09
Physical health	−0.42	0.06	(−0.55, −0.28)	−0.24	−60.31	0.000	
Weekly time invested	−0.30	0.06	(−0.42, −0.18)	−0.19	−40.97	0.000	
Model 3:							0.09
Physical health	−0.35	0.07	(−0.50, −0.21)	−0.20	−40.83	0.000	
Weekly time invested	−0.29	0.06	(−0.41, −0.17)	−0.18	−40.83	0.000	
Psychological	−0.11	0.06	(−0.23, −0.00)	−0.08	−10.97	0.048	
